# Middle-Scale Ionospheric Disturbances Observed by the Oblique-Incidence Ionosonde Detection Network in North China after the 2011 Tohoku Tsunamigenic Earthquake

**DOI:** 10.3390/s21031000

**Published:** 2021-02-02

**Authors:** Jin Wang, Gang Chen, Tao Yu, Zhongxin Deng, Xiangxiang Yan, Na Yang

**Affiliations:** 1 Institute of Geophysics & Geomatics, China University of Geosciences (Wuhan), Wuhan 430074, China; wangjin1221@whu.edu.cn (J.W.); yutao@cug.edu.cn (T.Y.); yanxx@cug.edu.cn (X.Y.); yangna@cug.edu.cn (N.Y.); 2 Electronic Information School, Wuhan University, Wuhan 430072, China; 3 The China Research Institute of Radio Wave Propagation (CRIRP), Qingdao 266107, China; dengzx@crirp.ac.cn

**Keywords:** ionosphere, gravity waves, middle-scale ionospheric disturbances, oblique-incidence ionosonde detection network, far-field inland propagation

## Abstract

The 2011 Tohoku earthquake and the following enormous tsunami caused great disturbances in the ionosphere that were observed in various regions along the Pacific Ocean. In this study, the oblique-incidence ionosonde detection network located in North China was applied to investigate the inland ionospheric disturbances related to the 2011 tsunamigenic earthquake. The ionosonde network consists of five transmitters and 20 receivers and can monitor regional ionosphere disturbances continuously and effectively. Based on the recorded electron density variations along the horizontal plane, the planar middle-scale ionospheric disturbances (MSTIDs) associated with the 2011 Tohoku tsunamigenic earthquake were detected more than 2000 km west of the epicenter about six hours later. The MSTIDs captured by the Digisonde, high-frequency (HF) Doppler measurement, and Constellation Observing System for Meteorology, Ionosphere, and Climate (COSMIC) satellite provided more information about the far-field inland propagation characteristics of the westward propagating gravity waves. The results imply that the ionosonde network has the potential for remote sensing of ionospheric disturbances induced by tsunamigenic earthquakes and provide a perspective for investigating the propagation process of associated gravity waves.

## 1. Introduction

The Tohoku earthquake (Mw = 9.0) occurred at 05:46:24 UT on 11 March 2011 near the east coast of Honshu, Japan (38.297° N, 142.373° E), as estimated by the US Geological Survey (USGS), followed by a powerful tsunami and several aftershocks. Vertical crustal movement during a tsunamigenic earthquake can induce fluctuation of the atmosphere on the surface of the ground and ocean, thus producing upward propagating atmospheric waves. Due to the conservation of kinetic energy, these waves grow nearly exponentially with decreasing atmospheric density, and at ionospheric heights, their amplitude is strong enough to be observed by a radio system [1,2]. Activated atmospheric waves can interact with ionized gas in the ionosphere and manifest themselves as seismotraveling ionospheric disturbances (STIDs) [3,4].

The horizontal and vertical propagation of STIDs triggered by the 11 March 2011 Tohoku earthquake and the subsequent tsunami near the east coast of Japan have been observed by various atmospheric and ionospheric observation instruments, including ionosonde [5,6,7,8], global positioning system (GPS) network [9,10,11,12], all-sky airglow imager [13,14,15], high-frequency (HF) Doppler measurements [16,17], and satellite/occultation observations [4,18,19,20,21]. The STIDs associated with the 2011 Tohoku tsunamigenic earthquake along the open sea (Pacific Ocean) have been widely reported using GPS network and all-sky airglow observations. Smith et al. [15] analyzed the all-sky imaging at the EI Leonciti Observatory, Argentina, and found that the thermospheric gravity waves associated with the Mw9.0 Tohoku (Japan) tsunamigenic earthquake propagated nearly 18,000 km away from the epicenter. Crowley et al. [3] analyzed GPS observations throughout continental United States and proposed that the STIDs following the 2011 Tohoku tsunami not only propagated to the U.S. west coast across the open sea but also continued to propagate into inland for more than 1500 km.

However, the westward propagating STIDs were mainly recorded on the near-field over Japan. The dense GPS network located in Japan recorded the middle-scale concentric ring of the total electron content (TEC) enhancements and depletions propagating radially in all directions [9]. As suggested by Tsugawa et al. [22], these waves could propagate more than 1500 km away from the ionospheric epicenter with less dissipation and appeared in the western part of Japan until ~09:00 UT. Jin et al. [11] suggested that these postseismic ionospheric disturbances were related to both the main shock and the aftershocks, and three kinds of disturbances associated with Rayleigh wave, acoustic wave, and tsunami-generated waves were identified after the tsunamigenic earthquake. Later, Chen et al. [7] proposed that the ionospheric waves induced by the 2011 Tohoku tsunami propagated over Japan and Korea and finally reached areas of China with a much slower horizontal speed (111 m/s). However, there have been a few reports about the far-field horizontal characteristics of the westward traveling ionospheric disturbances induced by the earthquake/tsunami displacement.

In this study, the dense oblique-incidence ionosonde detection network (OIIDN) located in North China was applied to extend the spatial measurement near Japan. The network consists of many separable transmitters and receivers operated in the HF band and can receive the echoes reflected from the ionosphere. Similar to the traditional vertical-incidence (VI) ionosonde, the oblique-incidence (OI) ionosonde has high sensitivity for electron density variations. Its multitransmitter and multireceiver operating modes provide an opportunity to further study the far-field horizontal propagation characteristics of the ionospheric disturbances after the 2011 Tohoku tsunamigenic earthquake.

## 2. Observation Network Description

**Figure 1:**
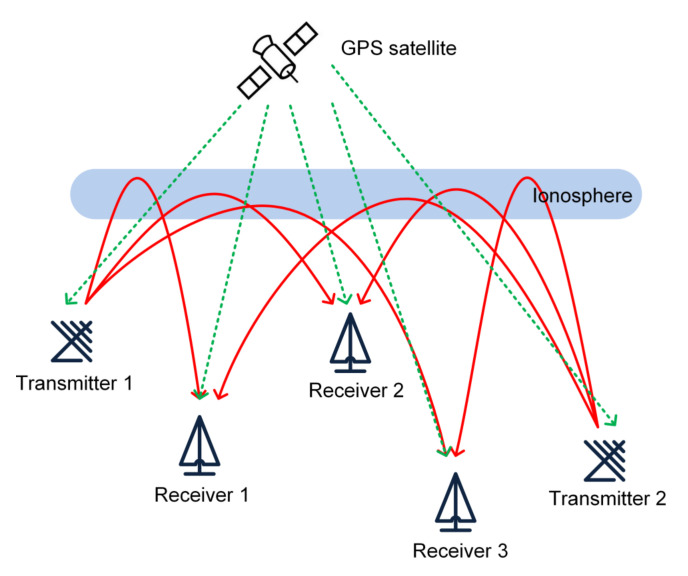
Simplified diagram of the working principle of an oblique-incidence ionosonde detection network composed of two transmitters and three receivers. The global positioning system (GPS) satellite provides time and frequency synchronization signals to all the transmitters and receivers.

The ionosonde network located in North China was developed by the China Research Institute of Radio Wave Propagation (CRIRP). It consists of five transmitters and 20 receivers and operates in the HF band ranging from 3 to 25 MHz. Figure 1 displays a simplified diagram of the operating principle of an OI detection network consisting of two transmitters and three receivers. The GPS satellite provides time and frequency synchronization signals to the transmitters and receivers. The transmitters transmit the radio waves, and the receivers receive the echoes reflected from the ionospheric E- and F-regions. Usually, the ionospheric reflection point is considered as the midpoint between the transmitter and the receiver.

**Figure 2:**
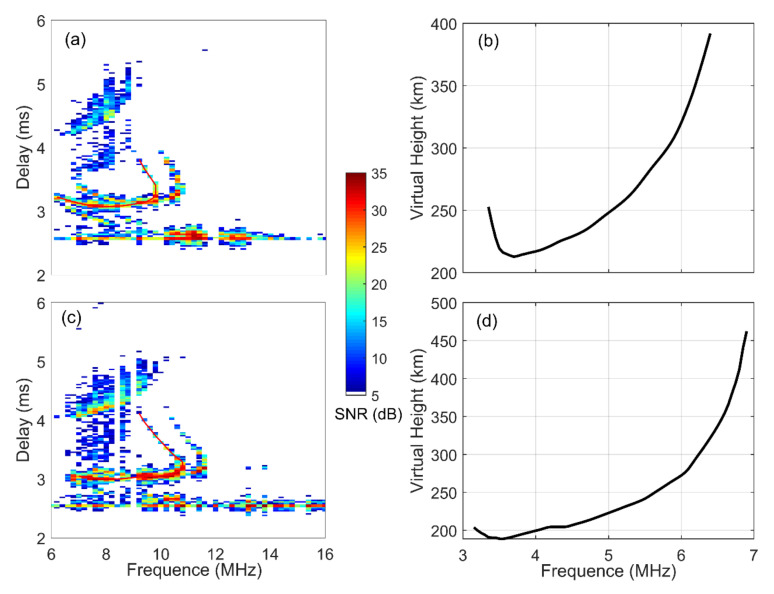
Oblique-incidence ionograms and the corresponding inversed vertical echo traces. (**a**,**c**) The recorded oblique-incidence ionograms. (**b**,**d**) The inversed vertical echo derived from the ordinary wave trace that were reflected from the ionospheric F2 layer (red curve).

Based on the inversion algorithm, OI ionograms can be inverted into the corresponding VI ionogram, as shown in Figure 2 [23,24]. The steps are as follows. First, the ordinary echoes of the OI ionogram are traced as the red curves. Then, based on the Martyn’s equivalent path theorem with the assumptions that the ionosphere is spherically stratified and the effect of geomagnetic field can be ignored, the inverted VI trace is obtained, as shown in Figure 2b,d [25]. Thus, the typical ionospheric parameters, such as the F2 layer critical frequency (*f*_o_F2) and the F2 layer bottom height (*h*′F2), can be estimated from the inverted VI trace. The five transmitters operate in the time-sharing mode, and the operating period of each transmitter is 30 min. Thus, the network can provide ~100 OI ionograms every 30 min.

**Figure 3:**
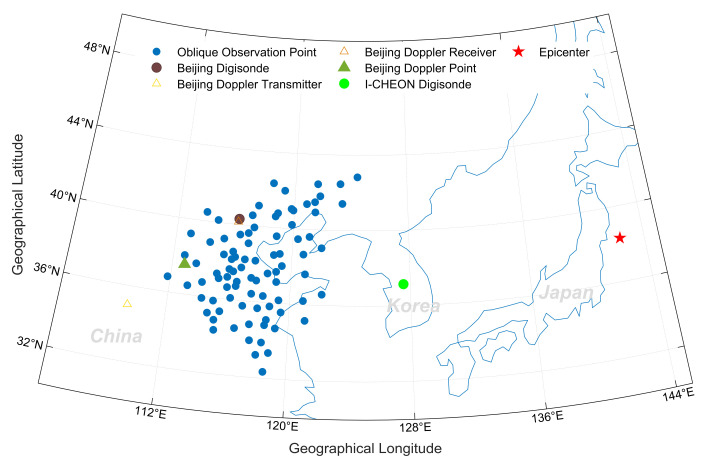
Map of East Asia showing the epicenter of the earthquake that occurred on 11 March 2011 (red star) and the ionospheric observation points of the ionosonde network (blue dots). The green solid dot represents the I-CHEON Digisonde. The dark-brown dot represents the Beijing Digisonde, and the cyan-blue solid triangle represents the observation point of the Beijing high-frequency (HF) Doppler. The light-brown and dark-brown hollow triangles represent the Doppler transmitter and receiver, respectively.

According to the location of the transmitters and receivers, the ionospheric observation points of the network were calculated and are shown in Figure 3 as blue dots. Some receivers were not in operation on that day, so 87 observation points were applied in this study. The corresponding observation area ranged from 111.82 to 124.43° E longitude and 32.34 to 42.84° N latitude. The maximum zonal and meridian distances of the observation region of the OIIDN were 808.09 and 1039.03 km, respectively. The distance between the most adjacent observation points was less than 50 km; thus, it had enough spatial resolution to recognize waves in the ionosphere with wavelengths greater than 100 km in the horizontal direction. Besides the ionosonde network, two other kinds of ground-based instruments were also employed: one was the Digisonde located in Beijing (40.25° N, 116.19° E) and I-CHEON (37.14° N, 127.54° E), and the other was Beijing HF Doppler sounding. The Beijing Digisonde can record VI ionograms every 15 min, and the I-CHEON Digisonde has the time resolution of 7.5 min. They can provide ionospheric parameters such as *f*_o_F2, *h*′F2, the peak height of the F2 layer (*h*_m_F2), and vertical plasma density profile. The HF Doppler receiver in Changping, Beijing (40.3° N, 116.2° E), was developed by Peking University. It can measure 10 MHz stabilized frequency radio waves transmitted from the National Time Service Center (NTSC) (35.0° N, 109.5° E) with a 10 s sampling period [16,26,27]. The recorded Doppler shift represents ionospheric variations at the midpoint (37.65° N, 112.85° E, cyan-blue solid triangles in Figure 3) between the NTSC and the receiver.

## 3. Data Analysis Method

To exclude the effect of day-to-day variations, the differential *f*_o_F2 (∆*f*_o_F2) recorded by each receiver in the network was calculated by the following equation:

∆*f*_o_F2 = (*f*_o_F2_e_ − *f*_o_F2_m_)/*f*_o_F2_m_ (%) (1)

where *f*_o_F2_e_ represents the electron density variations recorded on 11 March 2011, and *f*_o_F2_m_ denotes the monthly mean of the *f*_o_F2 value except on the earthquake day. This is a traditional method to remove day-to-day *f*_o_F2 variations. With the two-dimensional (2D) kriging interpolation method, the ∆*f*_o_F2 values observed at 88 ionospheric observation points (87 oblique observation points added to the Beijing Digisonde as mentioned above) were used to compose ∆*f*_o_F2 maps within the longitude range of 110 to 125° E and latitude range of 30 to 45° N. The kriging interpolation method was applied in this study to obtain two-dimensional (2D) ionospheric variations in the coverage areas of the OIIDN. As shown in Figure 3, the ionospheric observation points of the network are fairly dense and uniformly distributed, so fairly good estimations can be available for most of the observation area [28].

## 4. Results and Discussions

### 4.1. Horizontal Propagation Characteristics of the Disturbances

**Figure 4:**
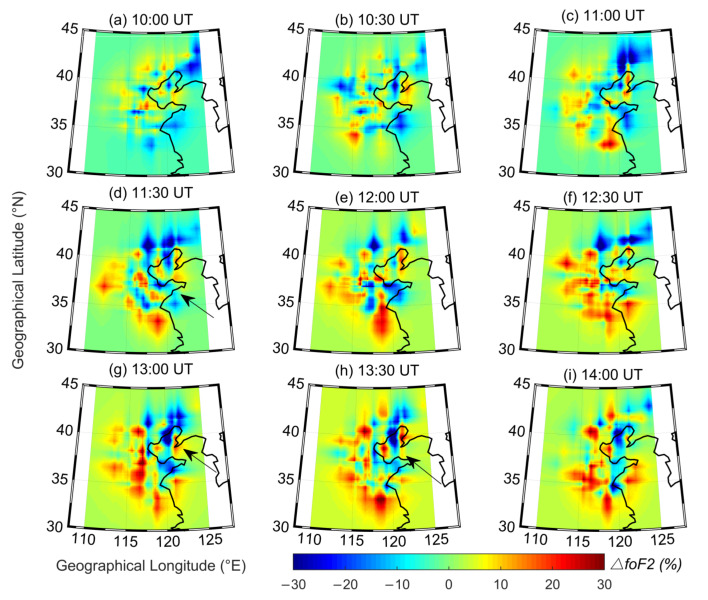
(**a**–**i**) Time sequences of two-dimensional ∆*f*_o_F2 maps recorded from 10:00 UT to 14:00 UT on 11 March 2011 with a 30 min step. The black curve in each plot is the coastline of East Asia. The colorbar represents the magnitude of the ∆*f*_o_F2 variations. The arrows indicate the ionospheric disturbances in the map. UT = LT − 8 h.

Figure 4 shows the 2D ∆*f*_o_F2 maps recorded by the ionosonde network every 30 min after the 2011 Tohoku tsunamigenic earthquake during the period from 10:00 to 14:00 UT. In the nine panels, the electron density fluctuations occurred during the whole observation period. However, the ∆*f*_o_F2 variations did not present obvious regularity until 11:30 UT (UT = LT − 8 h); at that time, the variations of the electron density showed a clear planar wave with enhancements and depletion strips alternating (Figure 4d). At 12:00 UT in Figure 4e, the planar wave twisted and disappeared. Soon afterward, a similar planar wave appeared again in the northeast of the observation area at 13:00 UT (Figure 4g). Then, the waves developed and covered most of the observation area at 13:30 UT and disappeared at 14:00 UT (Figure 4h,i). The duration of the planar waves observed by the network was approximately 0.5 to 1 h, which is similar to the simulation results of Coïsson et al. [29]. They suggested that the duration of electromagnetic waves being significantly disturbed by tsunami-induced gravity waves was approximately 40 min. The 2D ∆*f*_o_F2 maps that recorded during the period of 10:00–14:00 UT on 9 and 10 March 2011 are also displayed in Figures S1 and S2 (see Supplementary Materials), respectively. However, the ∆*f*_o_F2 is fluctuated without regularity and no similar ionospheric disturbances were observed two days before.

**Figure 5:**
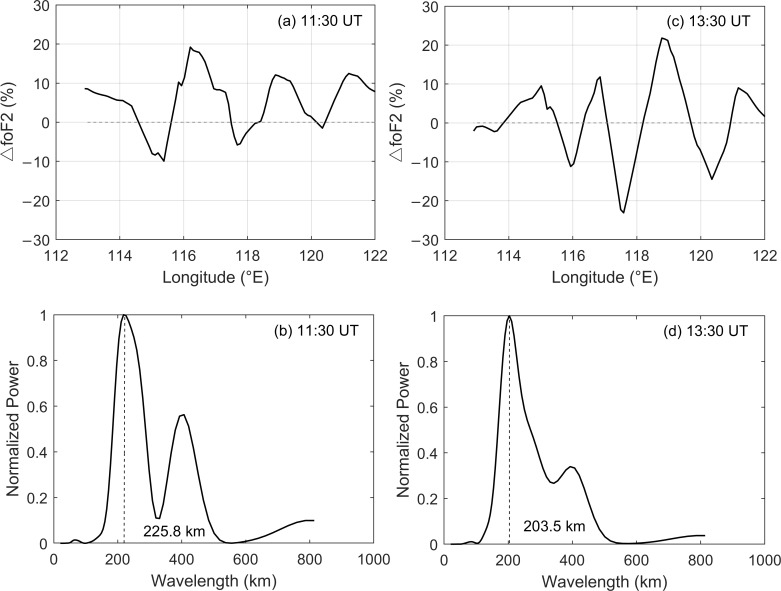
Variations of the ∆*f*_o_F2 variations along 38° N latitude from 113 to 122° E longitude and the corresponding horizontal wavelength of the recorded waves. The upper panels display the ∆*f*_o_F2 variations observed at (**a**) 11:30 UT and (**c**) 13:30 UT. (**b**,**d**) The relevant spectrum analysis results. The estimated wavelength is indicated by the vertical dotted line.

To further study the two-dimensional horizontal characteristics of the recorded ionospheric disturbances, the relative ∆*f*_o_F2 variations at 38° N (near the latitude of the epicenter) at 11:30 and 13:30 UT in the longitude coordinate are presented in the first panel of Figure 5 and the corresponding spectra are displayed on the bottom row. It can be seen that the ∆*f*_o_F2 increased and decreased alternately along the longitude (Figure 5a,c). The wavelength spectra revealed that the horizontal wavelengths of the recorded waves were 225.8 and 203.5 km, respectively (Figure 5b,d). The measured horizontal wavelengths in this study were similar to the GPS network [30,31] and all-sky airglow imager [13] observations as well as the related simulations [32]. These suggested that the horizontal wavelength of the gravity waves following the 2011 Tohoku tsunamigenic earthquake ranged from 200 to 400 km. Yasyukevich et al. [12] also indicated that the wavelength of the gravity waves induced by the 2011 Tohoku earthquake was ~200 km.

**Figure 6:**
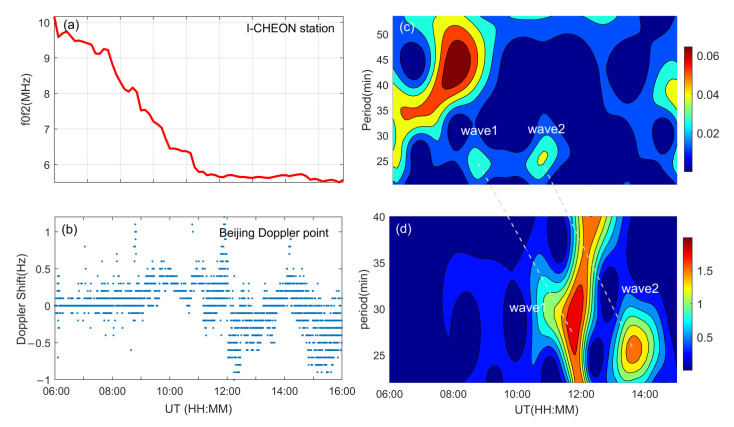
Variations of the *f*_o_F2 and Doppler shift and their spectrum analysis on 11 March 2011. (**a**) Ionospheric *f*_o_F2 variations recorded by the I-CHEON Digisonde and (**c**) its time-period spectrum. (**b**) Variations of the Doppler shift recorded by the Beijing Doppler receiver and (**d**) its temporal spectrum.

Due to the 30 min temporal resolution, the waves with periods less than 1 h cannot be recognized by the ionosonde network. On the other hand, the sampling rate of the Beijing HF Doppler receiver is 10 s [16], and the disturbances of the smaller periods can be observed. The recorded ionospheric Doppler shift variations are displayed in Figure 6b. It can be seen that the Doppler shifts exceeded 1 Hz several times. The wavelet analysis of the Doppler shift in Figure 6d implies that two waves were also recorded by the HF Doppler measurement. One appeared from 11:34 to 12:02 UT with period of 25.3–32.3 min, and the other appeared from 13:24 to 13:53 UT with period of 24.4–27.2 min. It can be noted that the ionospheric periodic disturbances recorded by the Doppler receiver were slightly later than the waves recorded by the network because the Doppler observation point is farther away from the epicenter than the ionosonde network. Similar ionospheric disturbances were also observed by the I-CHEON Digisonde (Korea) located between the epicenter (near Japan) and the OIIDN (China). The *f_o_*F2 variations measured by the Digisonde are presented in Figure 6a, and the corresponding time-period spectrum is shown in Figure 6c. Two waves with periods around 24.2 and 25.7 min were recorded at 8:15–8:45 and 10:15–10:45 UT, respectively, as shown in Figure 6c. By comparing the waves in the two spectra in the right column of Figure 6, it can be seen that the two instruments recorded the same waves at different times as indicated by the two dashed lines. According to the occurrence time of the waves recorded by the Digisonde, ionosonde network, and Doppler receiver, we deduced that the waves traveled westward across Korea and reached North China. It is very likely that the waves were produced by the 2011 Tohoku tsunamigenic earthquake.

Accordingly, combined with the horizontal wavelength and period of the waves as analyzed above, the horizontal velocities of the two wave components were estimated as 116.5–148.7 and 124.7–139.0 m/s, respectively; thus, they belong to middle-scale ionospheric disturbances (MSTIDs).

It is well known that three kinds of ionospheric disturbances were observed after the 2011 Tohoku tsunamigenic earthquake related to the source displacement, the Rayleigh waves, and the tsunami waves. The three kinds of ionospheric disturbances have different propagating speeds, which are associated with seismic Rayleigh waves (2.0–3.0 km/s), acoustic waves (0.3–1.5 km/s), and gravity waves (0.1–0.3 km/s) [5,9,11,12,30,33]. As studied by Galvan et al. [30] and Jin et al. [11], the most disturbed region after the 2011 tsunamigenic earthquake was located in the northwest part of the epicenter. They suggested that the related STIDs could propagate more than 1500 km with less dissipation and appeared in the western part of Japan a few hours later [22]. Therefore, we conjectured that the MSTIDs recorded by the ionosonde network were much likely attributed to the westward traveling gravity waves following the 2011 Tohoku tsunamigenic earthquake. The large vertical motions around the epicenter can generate acoustic (pressure) and gravity (buoyancy) waves in the neutral atmosphere, which can propagate upward into the ionosphere, interact with the ionized gas, and disturb the ionospheric plasma in the form of TIDs. Matsumura et al. [32] also suggested that these northwestward TIDs were partially driven by the earthquake through a direct excitation mechanism, i.e., the original displacement of the ocean surface produced the gravity waves associated with the earthquake. However, it is plausible that the westward portion of the tsunami waves also generated westward atmospheric gravity waves before reaching the Japan coastline. The gravity waves could have continued to propagate across Japan to some distances and result in the observed TIDs [5,7,11,30].

As suggested by Savastano et al. [34], the relationship between the detected TIDs and their sources can be proven by comparing the horizontal speed, direction, and spectral bandwidth of the waves with the same parameters of the possible source. In this study, the recorded MSTIDs showed that the waves had a period of 24–33 min, horizontal velocity of 116.0–149.0 m/s, and wavelength of 200–226 km near 11:30 and 13:30 UT. These parameters agree with the observations of Chen et al. [7] and Jin et al. [11] as well as the simulations of Occhipinti et al. [14] and Yu et al. [35]. The horizontal speed of the tsunamis approximately followed the shallow water wave speed $v=\sqrt{gh}$, where g is the gravitational acceleration and h is the ocean depth. Unlike the southeastward tsunami waves, the horizontal speed of the westward propagating STIDs caused by the westward Tohoku tsunami waves were much slower (~121 m/s) due to the approach of the Japan coastline (decrease of the ocean depth). Occhipinti et al. [14] simulated the propagating speeds of gravity waves in the northwest direction and showed that the speed decreased from ~300 to ~120 m/s with the increase of the propagating distance after the 2011 Tohoku tsunamigenic earthquake. Garcia et al. [18] also observed similar STIDs near areas in Southern China five hours after the 2011 Tohoku tsunamigenic earthquake. They showed that the speed and wavelength of the STIDs were 116 m/s and 244 km, respectively. Moreover, using the coastal tide gauges and bottom pressure stations, Shevchenko [36] considered that the low-frequency waves (~32 min period) from the 2011 tsunami could have existed for a long time in the northwest direction, which could have acted as the source of the STIDs. Liu et al. [4] also suggested that the ionosphere could have been disturbed by the 2011 Tohoku tsunami for several hours with much slower horizontal velocity (60–180 m/s) because the sea surface was continuously disturbed for more than 10 h after the tsunami occurrence. However, vertical displacement of the ocean surface induced by several aftershocks, as well as the additional turbulization in the upper atmosphere induced by a series of weaker temblors, can also generate significant gravity waves in the northwestward direction [12,22,30,32]. Future studies are needed to further investigate the source mechanism for the STIDs following the 2011 Tohoku tsunamigenic earthquake.

It is also worth noting that the planar waves were observed only in part of the observation areas (35–42° N latitude, 115–123° E longitude, shown in Figure 4d,h). Several reasons may be responsible for this. Firstly, detection of gravity waves in the ionosphere largely depends on the anisotropic coupling process between the neutral atmosphere and the charged ions controlled by the magnetic field orientation [37]. Secondly, the modulation effect of neutral winds, heat conduction, chemical reactions, and bathymetric effects can result in anisotropic and nonlinear ionospheric behavior of gravity waves [10,38,39,40,41]. Moreover, the relatively sparse observation points in the map would also result in deformation of the observed disturbances. 

The high geomagnetic activity on the earthquake day may have some influence on the study of ionospheric disturbances after the 2011 Tohoku tsunamigenic earthquake. However, as studied by Heki and Enomoto [42], ionospheric disturbances generated by great geomagnetic activity are a global phenomenon and will not appear in only local areas, and the disturbances will propagate southward. Chen et al. [7] studied the far-field ionospheric disturbances after the 2011 Tohoku earthquake and showed that the STIDs observed after 06:00 UT had little relationship with the high magnetic index. Liu et al. [4] also suggested that the magnetic storm has had little influence on ionospheric disturbances since the 2011 Tohoku earthquake and tsunami occurred. In our study, the MSTIDs only appeared in part of the whole map in Figure 4 and propagated westward but not equatorward. This may imply that the recorded MSTIDs had nothing to do with geomagnetic activities.

### 4.2. Vertical Propagation Characteristics of the Disturbances

**Figure 7:**
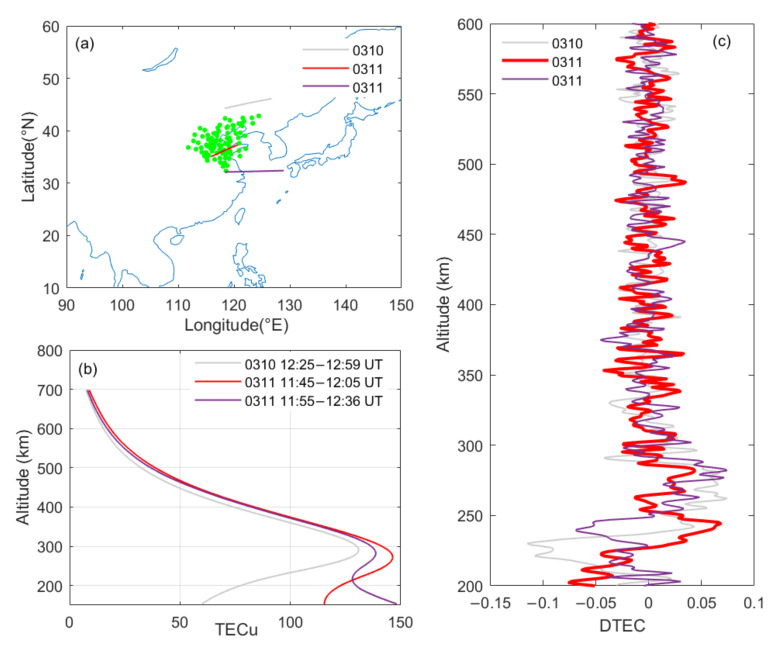
(**a**) Comparison of the total electron content (TEC) profiles observed before and near 12:00 UT around the ionosonde network. (**b**) The corresponding TEC profiles recorded by Constellation Observing System for Meteorology, Ionosphere, and Climate (COSMIC). (**c**) The detrended TEC profiles.

Coincidentally, the FORMOSAT-3/Constellation Observing System for Meteorology, Ionosphere, and Climate (COSMIC) crossed over/around the observation area of the network while the ionosonde network observed the STIDs. High-quality radio occultation (RO) profiles can be acquired by COSMIC with high vertical resolution and global coverage [43]. Thus, vertical propagation information of the observed waves can be achieved from the RO profiles. A comparative analysis of the TEC profiles recorded before and on the day of the 2011 Tohoku tsunamigenic earthquake near the ionosonde network near 12:00 UT is displayed in Figure 7. To reduce the influence of height discontinuity on the filtering effect, the recorded TEC profiles are interpolated to a height interval of 1 km (Figure 7b). The detrended TEC variations were obtained by removing the large-scale trends from the raw profiles (Figure 7c). The large-scale trends were obtained by least square regression with a 55-point moving window.

**Figure 8:**
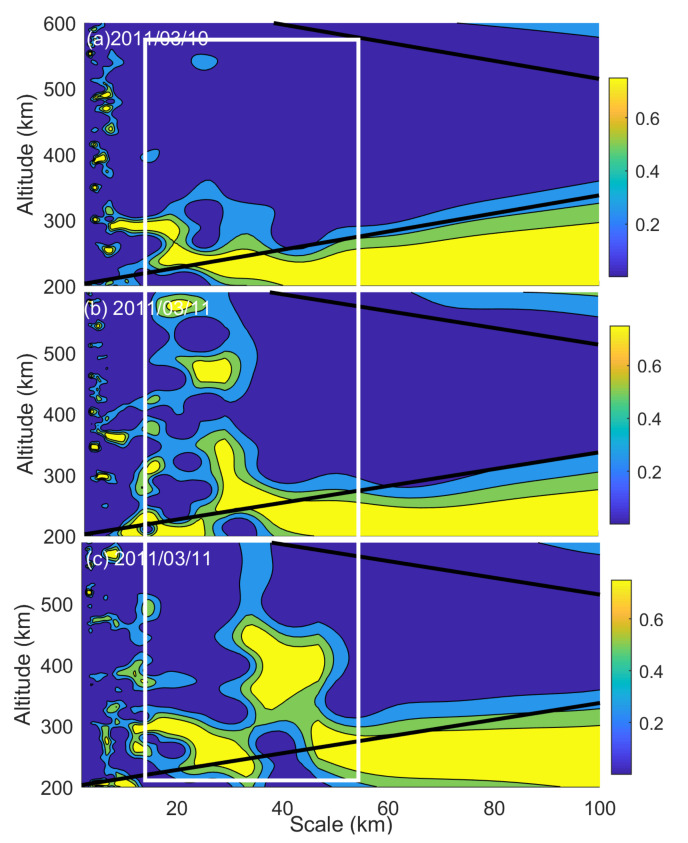
Wavelet analysis of the detrended total electron content (DTEC) profile variations on 10 and 11 March 2011. (**a**) The spectrum analysis of the DTEC on 10 March 2011. (**b**,**c**) The spectrum analysis of the DTEC variations recorded during 11:45–12:05 UT and 11:55–12:36 UT, respectively, on the earthquake day. The vertical white lines mark the time period when the ionospheric disturbances were observed. The black oblique lines indicate the cone of influence.

As revealed in Figure 7c, small-scale ionospheric fluctuations were recorded on both March 10 and 11, 2011, during 11:00–13:00 UT. Wavelet analysis was subsequently applied to further examine small-scale fluctuations on the TEC profiles (Figure 8). It can be clearly seen that TEC fluctuations with vertical wavelengths ranging from 10 to 40 km emerged and significantly enhanced on the earthquake day. According to the yellow contours, waves with vertical wavelengths of 29.8, 27.8, and 19.0 km appeared at altitudes of 331.6, 470.6, and 576.6 km, respectively. The results agree with the observations of Yan et al. [21] and Liu et al. [4]. They suggested that small-scale ionospheric fluctuations are often observed in daily observations but that the energy of these fluctuations will be significantly enhanced when an earthquake/tsunami occurs. Their results also showed that the vertical wavelengths of gravity waves in the ionosphere were mainly distributed between 10 and 45 km after the 2011 Tohoku tsunamigenic earthquake. Chen et al. [7] also suggested that the vertical wavelength of STIDs was close to 28 km and that the vertical group velocity and the center period of disturbance were 18.8 m/s and 25 min, respectively, after the 2011 Tohoku tsunami. As the COSMIC did not pass through the ionosonde network near 13:30 UT, no TEC profiles can be presented for that time.

### 4.3. Summary

The propagation characteristics of the recorded disturbances are listed in Table 1. The results reveal that the MSTIDs observed in North China were related to the far-field westward propagating gravity waves induced by the 2011 tsunamigenic earthquake. The Rayleigh waves and acoustic waves may be excluded considering their relatively fast propagating speed of kilometer magnitude and their small oscillation periods (less than 10 min) [16,33,44]. Regarding some differences in the parameters of the observed waves compared with previous studies, this may have resulted from the different observation measurements and calculation methods [12].

The agreement between our observations and the previous studies reveal that the oblique-incidence ionosonde network can be used to observe ionospheric disturbances associated with an tsunamigenic earthquake. The observation also provides preliminary information about the propagation and morphological characteristics of the far-filed ionospheric disturbances following the 2011 Tohoku tsunamigenic earthquake. This would be helpful for advancing our understanding of the tsunami/earthquake atmosphere–ionosphere coupling process in the future.

## 5. Conclusions

We studied the gravity waves propagated far westward from the source of the 2011 Tohoku tsunamigenic earthquake. The oblique-incidence ionosonde detection network combined with Digisonde, HF Doppler, and COSMIC observations detected middle-scale ionospheric disturbances with a horizontal wavelength of 200–226 km, period of 24–33 min, and speed of 116.0–149.0 m/s. The observed disturbances in North China, located more than 2000 km away from the epicenter, illustrates how efficiently wave energy can be transported. This study reveals the remote sensing ability of the ionosonde network for ionospheric disturbances caused by tsunamigenic earthquakes and provides a new opportunity in ionospheric tsunami seismology.

## Figures and Tables

**Table 1:** Propagation parameters of the recorded waves.

Time	11:30 UT	13:30 UT
Horizontal wavelength	225.80 km	203.50 km
Period	25.30–32.30 min	24.40–27.20 min
Horizontal speed	116.5–148.7 m/s	124.7–139.0 m/s
Vertical wavelength	19–50 km	—

## Data Availability

The Doppler Receiver and Digisonde data is available at https://data.meridianproject.ac.cn/. The COSMIC data is available at http://cdaac-www.cosmic.ucar.edu/cdaac/index.html. The Oblique-Incidence Ionosonde Detection Network data are available on request from the corresponding author. The data are not publicly available due to the project requirement.

## Supplementary Materials

The following are available online at https://www.mdpi.com/1424-8220/21/3/1000/s1, Figure S1: (**a**–**i**) Time sequences of two-dimensional ∆*f*_o_F2 maps recorded from 10:00 UT to 14:00 UT on 9 March 2011 with a 30-min step, Figure S2: Same as Figure S1, but for the two-dimensional ∆*f*_o_F2 variations on 10 March 2011.
