# Supplementary material for: Middle-Scale Ionospheric Disturbances Observed by the Oblique-Incidence Ionosonde Detection Network in North China after the 2011 Tohoku Tsunamigenic Earthquake
Source: Sensors (Basel). 2021 Feb 2;21(3):1000. doi: 10.3390/s21031000 (PMC7867239; doi:10.3390/s21031000)
Supplement: Supplementary file 1 [file sensors-21-01000-s001.pdf]

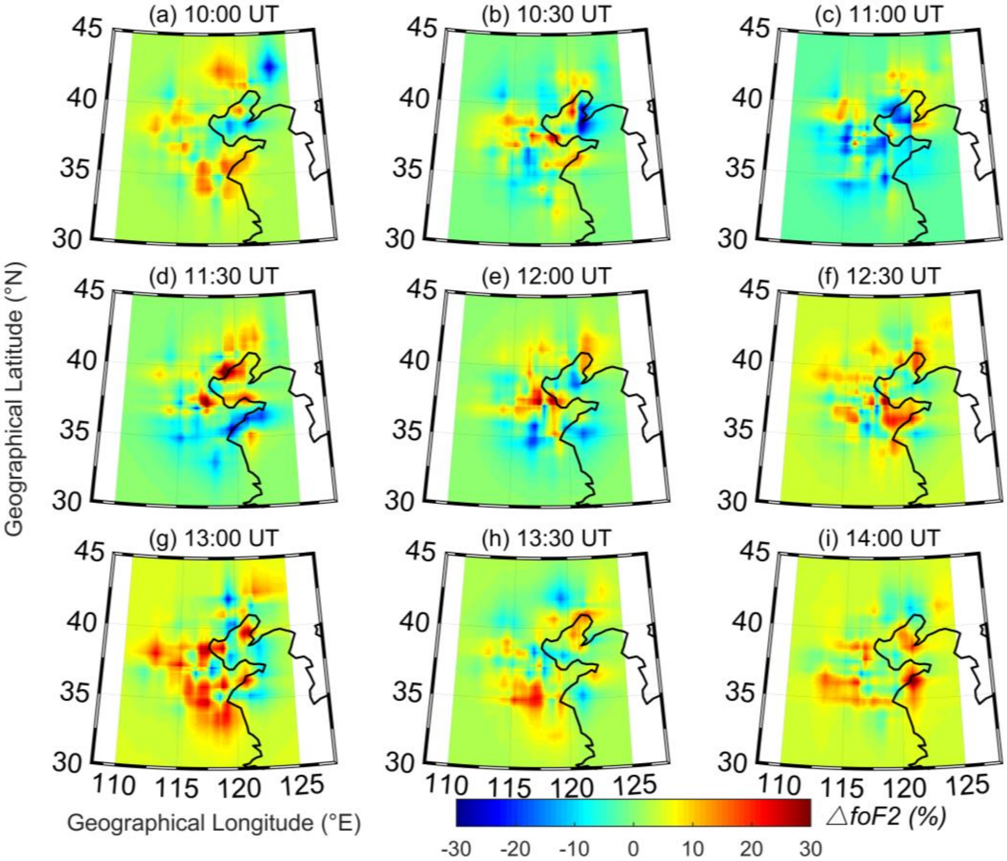

Figure S1. (**a**)–(**i**) Time sequences of two-dimensional Δf_oF2 maps recorded from 10:00 UT to 14:00 UT on 9 March 2011 with a 30-min step.

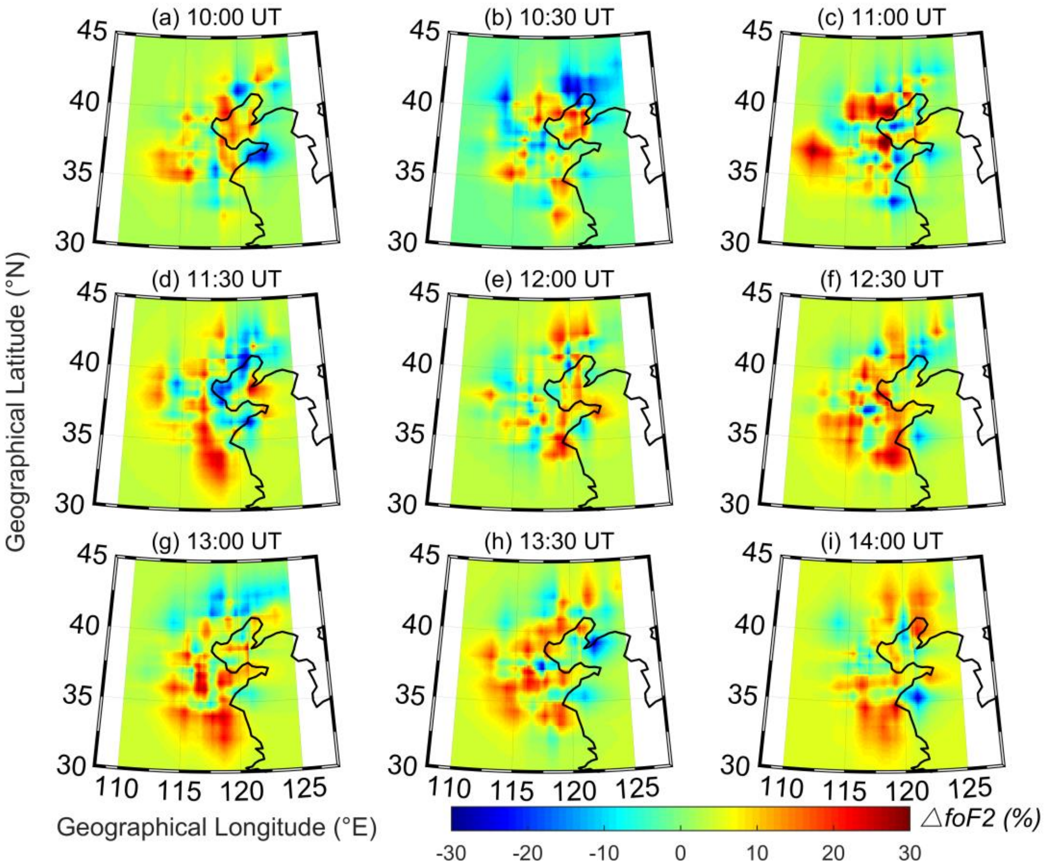

Figure S2. Same as Figure S1, (**a**)–(**i**) Time sequences of two-dimensional Δf_oF2 maps recorded from 10:00 UT to 14:00 UT on 9 March 2011 with a 30-min step, but for the two-dimensional Δf_oF2 variations on 10 March 2011.
